# Cystatin C at Admission in the Intensive Care Unit Predicts Mortality among Elderly Patients

**DOI:** 10.5402/2013/673795

**Published:** 2013-10-24

**Authors:** Maria Aparecida Dalboni, Daniel de Oliveira Beraldo, Beata Marie Redublo Quinto, Rosângela Blaya, Roberto Narciso, Moacir Oliveira, Júlio César Martins Monte, Marcelino de Souza Durão, Miguel Cendoroglo, Oscar Fernando Pavão, Marcelo Costa Batista

**Affiliations:** ^1^Universidade Federal de São Paulo, Botucatu Street, n740, Vila Clementino, 04023-062 São Paulo, SP, Brazil; ^2^Hospital Israelita Albert Einstein, Albert Einstein Avenue, n627, Morumbi, 05652-900 São Paulo, SP, Brazil; ^3^Division of Nephrology, Tufts University, Boston Avenue, n419, Medford, MA 02155, USA

## Abstract

*Introduction*. Cystatin C has been used in the critical care setting to evaluate renal function. Nevertheless, it has also been found to correlate with mortality, but it is not clear whether this association is due to acute kidney injury (AKI) or to other mechanism. *Objective*. To evaluate whether serum cystatin C at intensive care unit (ICU) entry predicts AKI and mortality in elderly patients. *Materials and Methods*. It was a prospective study of ICU elderly patients without AKI at admission. We evaluated 400 patients based on normality for serum cystatin C at ICU entry, of whom 234 (58%) were selected and 45 (19%) developed AKI. *Results*. We observed that higher serum levels of cystatin C did not predict AKI (1.05 ± 0.48 versus 0.94 ± 0.36 mg/L; *P* = 0.1). However, it was an independent predictor of mortality, H.R. = 6.16 (95% CI 1.46–26.00; *P* = 0.01), in contrast with AKI, which was not associated with death. In the ROC curves, cystatin C also provided a moderate and significant area (0.67; *P* = 0.03) compared to AKI (0.47; *P* = 0.6) to detect death. *Conclusion*. We demonstrated that higher cystatin C levels are an independent predictor of mortality in ICU elderly patients and may be used as a marker of poor prognosis.

## 1. Introduction

It has been reported that critically ill elderly patients in the intensive care unit (ICU) have a higher risk of developing acute kidney injury (AKI) [1, 2]. Despite significant improvements in therapeutics, AKI remains one of the main risk factors that contribute to morbidity (distant organ injury, prolonged ICU, and hospital stay) and high mortality rate in this population [3–5].

A rapid decline of glomerular filtration rate (GFR) and an increase in serum creatinine are still routinely used to characterize AKI [6, 7], promoting a delay in its clinical diagnosis and less opportunity for therapeutic intervention before injury becomes more established. Thus, there is an urgent need to investigate new biomarkers to improve the early detection of renal function damage and avoid poor outcomes.

Recently, urinary biomarkers, such as interleukin-18 (IL-18), kidney injury molecule-1 (KIM-1), and neutrophil gelatinase-associated lipocalin (NGAL), have been used for the early detection of acute kidney injury [8]. However, these markers have the disadvantage of urine collection, which is costly and may delay the initiation and adjustment of the treatment of critically ill patients. Nowadays, some plasma biomarkers have also been proposed for the early diagnosis of the AKI and its clinical outcomes in a variety of clinical settings [9, 10].

Cystatin C is an endogenous 13 kDa nonglycosylated cysteine protease inhibitor produced by all nucleated cells at a constant rate, and unlike creatinine, it is unaffected by age, gender, muscle mass, or diet [11]. Besides, it is excreted by glomerular filtration, and there is no evidence of tubular secretion [12]. Recently, some reports have shown that both serum and urinary cystatin C predict AKI [13–16]. Koyner et al. reported in adult cardiothoracic surgery that serum cystatin C is superior to serum creatinine in the early diagnosis of AKI [17]. Another study has also suggested that cystatin C is a better marker of GFR compared to creatinine in critically ill patients [18]. Besides, recent report has found cystatin C to be a predictor of mortality independent of renal function [19]. In clinical practice, however, it is unclear what is the best independent marker to detect early AKI and whether it is associated with poor outcome in elderly ICU patients.

So, the aim of this study was to evaluate serum cystatin C level as a predictor of AKI occurrence and mortality in critically ill elderly patients.

## 2. Materials and Methods

This study was approved by the Universidade Federal de São Paulo and Hospital Israelita Albert Einstein Ethics Committee and was therefore in compliance with the Helsinki Declaration. Informed consent was obtained from all patients or their legal guardians, prior to enrollment.

### 2.1. Study Population

It was a prospective cohort study of critically ill elderly patients (>60 years old) without AKI at admission in ICU (initially normal serum creatinine levels). They were included on the first day of hospitalization in an ICU setting, and they were followed up prospectively during ICU stay to determine the occurrence of AKI and mortality. Patients not considered for resuscitation or kidney transplantation or with abnormal kidney function (abnormal serum creatinine levels) were excluded. The Acute Physiology and Chronic Health Evaluation Classification System II (*APACHE II) *score was used for quantifying the severity of the illness for the ICU patients [20, 21], and AKI was defined by Acute Kidney Injury Network (AKIN) criteria: serum creatinine increased 0.3 mg/dL or increased 1.5–2.0-fold from baseline or serum creatinine increased >2.0-3.0-fold from baseline or serum creatinine increased >3.0 fold from baseline or serum creatinine (≥4.0 mg/dL) with an acute increase of at least 0.5 mg/dL or needed for RRT [22].

All consecutive ICU patients were eligible for a prospective selection from February 2008 until April 2010. During this period, we included 400 ICU patients, of whom 234 were enrolled (normal serum creatinine), 45 (19%) developed AKI, and 189 did not develop renal dysfunction (control group) according to AKIN criteria. Epidemiological variables associated with AKI, such as sepsis, coronary artery disease, chronic heart failure, stroke, mechanical ventilation, use of vasopressor drugs, renal replacement therapy, chronic kidney disease, hypertension, and diabetes mellitus, were screened. We also evaluated the demographic and clinical characteristics of the patients according to normality for serum cystatin C at admission (≤0.96 and >0.96 mg/L).

We measured serum creatinine daily using the automated Jaffé method (CREA-Hitachi 912, Roche Diagnostics). Cystatin C was assessed only in the first 24 hours of ICU admission, and it was measured by nephelometry (Dade Behring, Marburg, Germany). Albumin was determined at admission by the colorimetric bromocresol green assay, C-reactive protein (CRP) at admission by nephelometry (Beckman, Galway, Ireland), and brain natriuretic peptide (BNP) at admission by a chemiluminescent method (ADVIA Centaur, Siemens, Berlin, Germany). Body mass index (BMI) was calculated for each patient.

### 2.2. Statistical Analyses

A statistical analysis was performed using the SPSS statistical software program (version 16.0, SPSS, Chicago, IL, USA). Continuous variables were expressed as means + SD or as percentages. Comparisons between groups were made by the chi-square test (*χ* ^2^) and Student's *t*-test, as appropriate.

Logistic regression analysis was used taking mortality as the dependent variable to evaluate cystatin C as an independent predictor for mortality, adjusted by age, gender, APACHE, use of vasopressor drugs, sepsis, ICU hospitalization days, and albumin.

The association between cystatin ≤0.96, cystatin >0.96 mg/L, AKI, and No AKI for risk of death was estimated by hazard ratios (HR), derived from the Cox proportional hazards regression model with adjustment for age and ICU diagnosis. The receiver operating characteristic (ROC) curves were generated for cystatin C and AKI to test the ability to detect death. Differences were considered statistically significant when two-tailed tests yielded *P* < 0.05.

## 3. Results

The demographics and clinical characteristics according to acute kidney injury (AKI) are shown in Table 1. We observed that those patients on mechanical ventilation were more prone to develop acute kidney injury (35.5%), and 17.8% of AKI patients needed renal replacement therapy during ICU stay. No other differences in the variables analyzed between the patients of two groups were achieved.

Higher serum levels of cystatin C were more prevalent in patients with higher serum levels of creatinine, in older patients, and those with more ICU hospitalization days (Table 2). Besides, higher serum levels of cystatin C at admission were associated with the development of sepsis (34.8% versus 13.8%, *P* < 0.001) and vasopressor drug use (23% versus 15%, *P* = 0.01).

**Figure 1:**
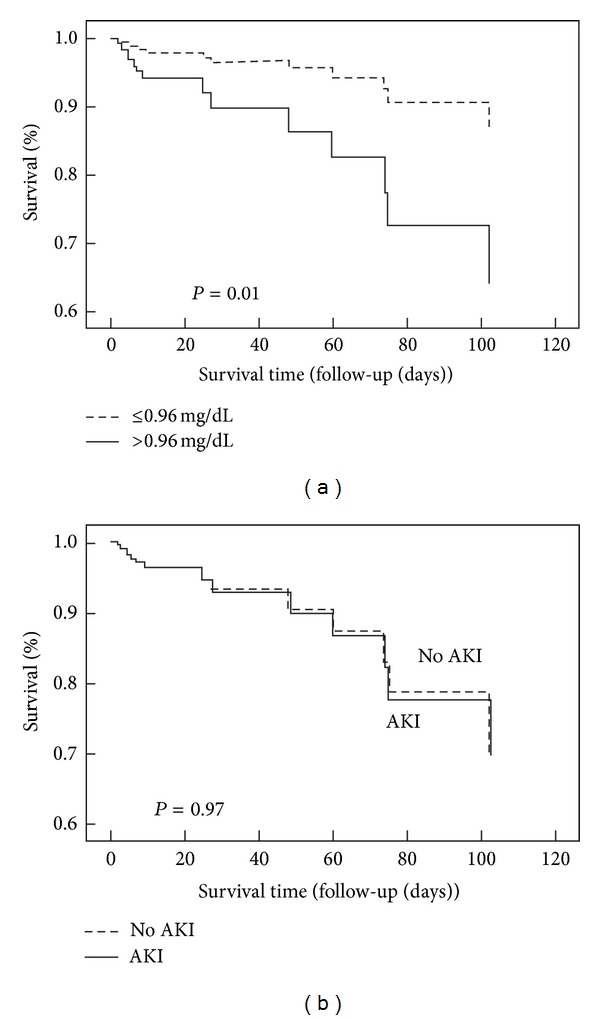
(a) Cumulative survival curves for risk of mortality stratified by cystatin C levels ≤0.96 and >0.96 mg/dL. (b) Cumulative survival curves for risk of mortality according to AKI and No AKI.

**Figure 2:**
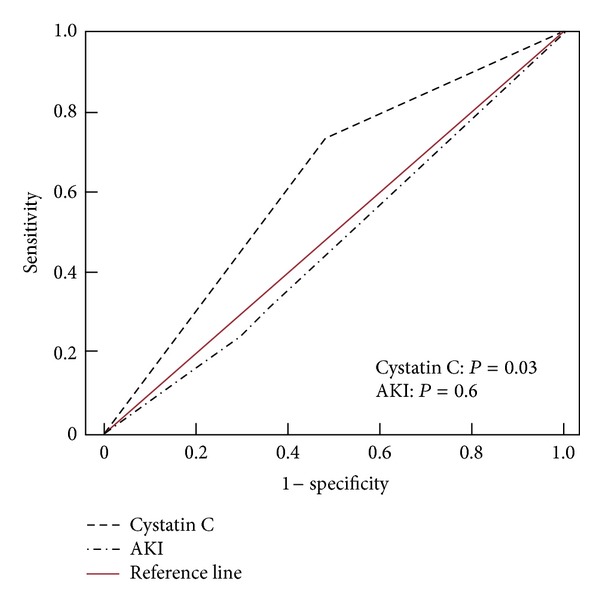
ROC Curve of Cystatin C and AKI as Markers to Death.

In logistic regression analysis with stepwise selection, adjusting for variables related to ICU mortality such as age, gender, APACHE II score, sepsis, vasopressor use, ICU hospitalization days, and albumin, we observed that an elevated cystatin C level was an independent predictor of mortality in our cohort (Table 3) (Figure 1). In contrast, AKI was not associated with mortality in the studied population (Figure 2).

In the ROC curves, cystatin C also provided a moderate and significant area (0.67; *P* = 0.03) compared to AKI occurrence (0.47; *P* = 0.6) to detect death (Figure 2).

## 4. Discussion

In this study we evaluated serum cystatin C as a predictor of AKI and mortality in critically ill elderly patients with normal serum creatinine at admission.

When we analyzed cystatin C with regard to AKI incidence, no significant difference was observed in the comparison between those with higher and normal cystatin C levels at admission. On the other hand, higher cystatin C level was an independent predictor of mortality in the ICU, when adjusted by age, gender, APACHE II score, vasopressors, sepsis, ICU hospitalization days, and serum albumin level. Wu et al. also reported that serum cystatin C wasassociated with death in an older population without AKI [23], and Carrasco-Sánchez et al. reported that cystatin C was a strong and independent predictor of an unfavorable outcome in patients with heart failure without renal dysfunction [24]. Despite that several authors mentioned that subjects with AKI have high mortality [2, 25, 26], we did not observe in our study this association.

The mechanisms involved in the relation between elevated cystatin C and mortality remain undetermined. Some authors speculate that cystatin C reflects the balance of its primary physiological determinants such as cellular generation, renal filtration, and consequent renal degradation [27]. Thus, an increased cystatin C concentration could identify early deviations in GFR and may play a sensitive indicator of “preclinical” renal disease, which may thus be associated with mortality [28].

In accordance withother studies, we observed that 19% of ICU patients developed AKI. However, cystatin C did not discriminate those patients who would prospectively develop AKI in our population. Perianayagam et al. also showed that cystatin C was inferior as predictor of AKI and dialysis requirement after adjustment to APACHE II, liver disease, sepsis, and mechanical ventilation in a cohort of 200 patients [29]. In contrast, Nejat et al. studied 442 critically ill ICU patients and 73 (37%) of whom developed AKI had increased plasma cystatin C before the increase in plasma creatinine [30]. These authors suggested that plasma cystatin C was superior to plasma creatinine as an early predictor of AKI, similar to findings previously described by Herget-Rosenthal et al. in the ICU population. Therefore, we can report that, in our cohort, a single measurement of cystatin C at admission and in a unique ICU setting does not discriminate AKI occurrence. Moreover, recent studies reported that cystatin C could reflect another pathogenic state affecting long-term outcome. These studies have demonstrated an increased baseline cystatin C in patients with HIV, cancer, corticosteroid treatment, and inflammation without renal failure or AKI occurrence [19, 31–33]. 

With regard to inflammation, we also observed in the present study an association between sepsis and high serum levels of cystatin C and a tendency of association between APACHE II and cystatin C. It could represent direct and indirect inflammations and, as suggested by some authors, it is possible that cystatin C may reflect pathogenic states other than GFR [34]. If this hypothesis is true, some of the associations between mortality and serum levels of cystatin C could reflect not only a loss of renal function but also an association between inflammation and mortality. Therefore, future case-control study in a larger sample size is necessary to prove this hypothesis.

Some problems have been reported in many studies when using cystatin C to evaluate GFR. The fact is that almost every study compares serum cystatin C with serum creatinine or estimated GFR, but neither is a reference method for GFR measurements. Yet, methods to determine cystatin C and creatinine vary. However, cystatin C has been reported to be a better marker of renal failure in older adults compared to serum creatinine, mainly in those with low creatinine, which could reinforce cystatin C as a marker not only of renal failure, but also may be an indicator of death in this population [35].

Finally, we showed that cystatin C is not a marker of acute kidney injury in elderly patients hospitalized in the intensive care unit when used AKIN as standard diagnostic criteria. On the other hand, it was an independent predictor of mortality in this population, contrary to what happened with the serum creatinine. Explanation for this combination of results is based on the possibility that serum cystatin C have been the really marker involved in the pathogenesis of acute kidney injury and, therefore, high values were associated with increased mortality. Serum creatinine, due to the chronic loss of muscle mass in the elderly and other critical factors already described, probably not determined appropriately renal function on admission and its evolution throughout the hospitalization (84), not allowing a correct discrimination of the groups (AKI and no AKI) on admission and consequently compromising the analysis of outcomes based on these groups.

## 5. Conclusions

We showed that serum cystatin C is an independent predictor of mortality in critically ill elderly patients, and it could be considered a marker of poor prognosis.

## Figures and Tables

**Table 1:** Demographic, clinical, and epidemiological characteristics of all patients according to acute kidney injury (AKI) (*n* = 234).

Characteristic	AKI (*n* = 45)	No AKI (*n* = 189)	*P *
Age (years)	74 ± 11	75 ± 11	0.89
BMI	26 ± 5	26 ± 5	0.96
Gender (% males)	70	60	0.13
ICU hospitalization (days)	7 ± 21	4 ± 6	0.11
APACHE II score	20 ± 5	18 ± 5	0.07
Albumin (mg/dL)*	3.00 ± 0.91	3.00 ± 0.60	0.82
CRP (mg/dL)*	9.00 ± 8.44	7.73 ± 8.77	0.38
sCr (mg/dL)*	0.80 ± 0.20	0.76 ± 0.22	0.35
Cystatin C (mg/L)*	1.05 ± 0.48	0.94 ± 0.36	0.10
Sepsis	9 (20%)	42 (22.2%)	0.84
CAD/heart failure	17 (37.7%)	70 (40.7%)	0.85
Stroke	4 (8.9%)	19 (10.1%)	0.82
Mechanical ventilation*	33 (35.5%)	52 (21.6%)	0.01
Use of vasopressor drugs	25 (26.9%)	53 (22.0%)	0.38
Hypertension	19 (42.2%)	99 (52.4%)	0.25
Diabetes mellitus	11 (24.4%)	48 (25.4%)	0.90
Death	3 (6.7%)	10 (5.3%)	0.72

Student's *t*-test; chi-square test (*χ* ^2^).

AKI: acute kidney injury, BMI: body mass index, ICU: intensive care unit, CRP: C-reactive protein, sCr: serum creatinine, CAD: coronary artery disease.

*At admission in ICU.

**Table 2:** Demographic and clinical characteristics of all patients according to serum levels of cystatin C ≤ 0.96 and >0.96 mg/L (*n* = 234).

Characteristic	Cystatin C ≤ 0.96 (*n* = 145)	Cystatin C > 0.96 (*n* = 89)	*P *
Age (years)	72 ± 11	78 ± 10	<0.01
BMI	26 ± 4	26 ± 6	0.59
Gender (% males)	60	58.4	0.89
ICU hospitalization (days)	4 ± 6	6 ± 16	0.04
APACHE II score	20 ± 5	18 ± 5	0.05
Albumin (mg/dL)*	3.09 ± 0.70	2.93 ± 0.57	0.07
CRP (mg/dL)*	7.60 ± 8.14	8.58 ± 9.57	0.40
sCr (mg/dL)*	0.74 ± 0.20	0.80 ± 0.24	0.03

Student's *t*-test.

BMI: body mass index, ICU: intensive care unit, CRP: C-reactive protein, sCr: serum creatinine.

*At admission in ICU.

**Table 3:** Logistic regression analysis of mortality according to cystatin C > 0.96 mg/L.

Outcome	HR	95% CI	*P* value
*Mortality *			
Unadjusted	6.11	1.66–22.5	0.007
Adjusted for age and gender	5.54	1.45–21.12	0.01
Adjusted for age, gender, and APACHE II score	5.35	1.39–20.51	0.01
Adjusted for age, gender, APACHE II score, and vasopressors	5.48	1.40–21.44	0.01
Adjusted for age, gender, APACHE II, vasopressors, and sepsis	5.00	1.26–20.11	0.02
Adjusted for age, gender APACHE II, vasopressors, sepsis, and ICU hospitalization	5.63	1.38–22.96	0.02
Adjusted for age, gender APACHE II, vasopressors, sepsis, ICU hospitalization, and albumin	6.16	1.46–26.00	0.01

HR: hazard ratio, ICU: intensive care unit.
